# Supplementary material for: Factors affecting utilization of outpatient healthcare services among the elderly population in Butiama and Musoma districts, Tanzania: A community-based cross-sectional study
Source: PLoS One. 2024 Jul 19;19(7):e0304687. doi: 10.1371/journal.pone.0304687 (PMC11259282; doi:10.1371/journal.pone.0304687)
Supplement: S1 Questionnaire — (DOCX) [file pone.0304687.s001.docx]

**Questionnaire (English version)**

**Research title:** “**Factors affecting utilization of outpatient healthcare services among the elderly population in Butiama and Musoma districts, Tanzania”: a community-based-cross-sectional study.**

**SOCIO-DEMOGRAPHIC INFORMATION**

| **S/N** | **Question** | **Option/response** | **Remark** |
| --- | --- | --- | --- |
| 01. | What is your age? | … |  |
| 02. | Sex (do not ask, just observe). | 1. Male  2. Female |  |
| 03. | What is your highest educational level? | 1. Unable to read and write  2. Primary school  3. Secondary school  4. College and above |  |
| 04. | What is your marital status? | 1. Single. 2. Married/ Co-cohabiting. 3. Divorced/Separated 4. Widowed. |  |
| 05. | What is your source | 1. Employed  2. Agriculture  3. Informal sector apart from agriculture  4. Homemaker |  |
| 06. | What is your monthly income? (Tsh) | 1. < 100,000  2. 100,000-500,000  3. 500,000-1,000,000  4. > 1,000,000 |  |
| 07. | How many family members are in your household? | 1. 1  2. 2  3. 3  4. ≥4 |  |
| 08. | Do you drink alcoholic beverages? | 0. Yes  1. No |  |
| 09. | Do you currently smoke cigarettes or use any tobacco products? | 1. Yes  2. No  3. Quitted |  |
| 10. | Do you do physical exercises regularly? | 0. Yes  1. No |  |
| 11. | Do you/your family own the following items;  1. Radio  2. TV  3. Mobile phone  4. Computer  5. Refrigerator  6. Bicycle  7. Animal-drawn cart  8. Motorcycle/Scouter  9. Car  10. Own none of the above | 0 = Lowest economic status (own none of the possessions or only radio),  1 = Second (radio, phone, bicycle),  2= Middle (radio, TV, mobile phone, bicycle)  3 = Fourth (radio, TV, mobile phone, refrigerator, bicycle, animal-drawn cart, motorcycle/scouter)  4 = Highest economic status (owns all in the list with a Car) |  |

**Perception on the utilization of healthcare services.**

Perception on healthcare utilization **(tick one)**

|  |  | **Strongly agree** | **Agree** | **Neutral** | **Disagree** | **Strongly**  **Disagree** |
| --- | --- | --- | --- | --- | --- | --- |
| **S/N** | **Questions** |  |  |  |  |  |
| 12 | Do you see the need for utilizing healthcare services? |  |  |  |  |  |
| 13 | Are you willing to utilize outpatient healthcare services? |  |  |  |  |  |
| 14 | Are you willing to be screened for different diseases without having any signs or symptoms of those diseases? |  |  |  |  |  |
| 15. | Do you fear the procedures used in the provision of healthcare services? |  |  |  |  |  |
| 16. | Do you feel shy about exposing private parts during the procedure to young or opposite-sex healthcare service providers? |  |  |  |  |  |
| 17. | Do you fear being diagnosed with the disease following screening processes? |  |  |  |  |  |
| 18. | Do you think the available healthcare services are expensive? |  |  |  |  |  |
| 19. | Like any elder, do you think you are susceptible to developing different diseases due to ageing processes? |  |  |  |  |  |
| 20. | Is it true healthcare providers are rude when providing care to patients? |  |  |  |  |  |
| 21. | Is it true early utilization of healthcare may be beneficial to health? |  |  |  |  |  |
| 22. | Do you think private hospitals have good health services compared to government-owned hospitals? |  |  |  |  |  |
| 23. | Do you think waiting to see doctors makes you feel like healthcare services are inconvenient? |  |  |  |  |  |
| 24. | What are your gender preferences for provider of healthcare services? |  |  |  |  |  |

25. General perception toward utilization of healthcare services.

0. Positive

1. Negative

**3. Level of utilization of outpatient health care services.**

26. In the last 12 months, have you ever visited a healthcare hospital to seek outpatient healthcare services? (If, no go to q 27)

0. Yes

1. No

27. In the last 12 months, how many times did you visit a healthcare facility for outpatient healthcare services?

1. Once

2. Twice

3. More than two times

28. What kind of hospital or establishment was that?

1. Public

2. Public

3. FBO

4. Don’t know

29. In the last 12 months have you ever felt you needed healthcare services but refrained from visiting a healthcare facility? (If yes, go to q30)

0. Yes

1. No

30. What reason(s) (if there were more than one) prevented you from getting medical attention?

1. The problem cleared up

2. Financial problem

3. Other reasons (mention……………………….)

I appreciate your time. Have you got any queries?

The hyperlink below is an English version of the questionnaire incorporated in the Kobo toolbox for data collection.

<https://ee.kobotoolbox.org/x/J9tq3sda>
