# Supplementary material for: Factors affecting utilization of outpatient healthcare services among the elderly population in Butiama and Musoma districts, Tanzania: A community-based cross-sectional study
Source: PLoS One. 2024 Jul 19;19(7):e0304687. doi: 10.1371/journal.pone.0304687 (PMC11259282; doi:10.1371/journal.pone.0304687)
Supplement: Attachment — Submitted filename: S1 rebuttal letter.docx [file pone.0304687.s004.docx]

**PONE-D-23-36486**

Factors affecting utilization of outpatient healthcare services among the elderly population in Butiama and Musoma Districts, Tanzania: A community-based cross-sectional Study

| **Editor** | **Quarries** | **Compliance in the manuscript** |
| --- | --- | --- |
|  | 1. Please ensure that your manuscript meets PLOS ONE's style requirements, including those for file naming. | Thank you for your valuable feedback. We have formatted the entire manuscript according to PLOS ONE's style requirements as requested. |
|  | 2. When completing the data availability statement of the submission form, you indicated that you would make your data available on acceptance. We strongly recommend all authors decide on a data-sharing plan before acceptance, as the process can be lengthy and hold up publication timelines. Please note that, though access restrictions are acceptable now, your entire data will need to be made freely accessible if your manuscript is accepted for publication. This policy applies to all data except where public deposition would breach compliance with the protocol approved by your research ethics board. If you are unable to adhere to our open data policy, please kindly revise your statement to explain your reasoning and we will seek the editor's input on an exemption. Please be assured that, once you have provided your new statement, the assessment of your exemption will not hold up the peer review process. | Thank you for your valuable feedback. We acknowledge your request and we commit to provide the entire dataset upon the manuscript's acceptance. |
|  | 3. PLOS requires an ORCID iD for the corresponding author in Editorial Manager on papers submitted after December 6th, 2016. Please ensure that you have an ORCID iD and that it is validated in Editorial Manager. | Thank you for your suggestion.  The ORCID iD for the corresponding author has already been supplied: it is 0009-0004-4332-3413. |
|  | 4. Please amend either the title on the online submission form (via Edit Submission) or the title in the manuscript so that they are identical. | Thank you for your comment.  The title has already been revised in the manuscript to match the information provided in the online submission form. |
|  | 5. We note you have included a table to which you do not refer in the text of your manuscript. Please ensure that you refer to Table 4 in your text; if accepted, production will need this reference to link the reader to the Table. | Thank you for bringing this to our attention.  We acknowledge the oversight and confirm that the necessary reference to Table 4 has been included in the revised manuscript. Page 12 |
|  | 6. Please review your reference list to ensure that it is complete and correct. If you have cited papers that have been retracted, please include the rationale for doing so in the manuscript text, or remove these references and replace them with relevant current references. Any changes to the reference list should be mentioned in the rebuttal letter that accompanies your revised manuscript. If you need to cite a retracted article, indicate the article’s retracted status in the References list and also include a citation and full reference for the retraction notice. | Thank you for your guidance.  Upon careful review, we confirm that each reference in our list is accurate and pertinent to the manuscript. We have ensured that there are no citations to retracted papers. No retracted articles are included in our reference list, and all citations align with relevant sources. There are no changes required in this regard for the revised manuscript.  In case of any specific concerns or further clarifications needed, please do not hesitate to let us know. |
|  | Additional Editor Comments: |  |
|  | 1. Please mention in the methods, how the wealth index was measured (any tool, or questionnaire-based, if questionnaire-based, which inputs were taken). | Thank you for your feedback.  The measurement of the wealth index has been addressed in the methods section of the manuscript. We have shared information about the utilized tool, which is questionnaire-based. Please refer to the supplementary file for details on Question 12 of the questionnaire.  The relevant inputs for the wealth index calculation have also been specified. If you have any additional questions or if further clarification is needed, please feel free to let us know. Available in manuscript page number 5 |
|  | 2. The recommendations section is more generic currently. Usually, the existing policies and policymakers do have a focus on the "super"-senior citizens (80 years and above), the elderly are already aware of the importance of health utilisation and those who are aware, do themselves take more active OPD visits. Instead, I suggest improving the recommendations based on your inferences such as "fear, shyness, gender sensitivity", etc. | Thank you for your insightful suggestion.  We appreciate your feedback on the recommendations section. The manuscript has been revised to enhance the specificity of recommendations. We believe these adjustments provide a more nuanced and context-specific set of recommendations. If you have any further recommendations or specific areas of concern, please feel free to let us know. Page number 16 |
