# Supplementary material for: Factors affecting utilization of outpatient healthcare services among the elderly population in Butiama and Musoma districts, Tanzania: A community-based cross-sectional study
Source: PLoS One. 2024 Jul 19;19(7):e0304687. doi: 10.1371/journal.pone.0304687 (PMC11259282; doi:10.1371/journal.pone.0304687)
Supplement: Attachment — Submitted filename: S1 rebuttal letter 1.docx [file pone.0304687.s005.docx]

**PONE-D-23-36486**

Factors affecting utilization of outpatient healthcare services among the elderly population in Butiama and Musoma Districts, Tanzania: A community-based cross-sectional Study

| S/N | **Comments from the reviewer** | **Response** | **Page no.** |
| --- | --- | --- | --- |
| 1. | “Longer life expectancies offer societal benefits but also bring complex challenges tied to an ageing population, impacting productivity, economic growth, and social security sustainability”. I would briefly specify some societal benefits and, as you state in the following paragraphs, I would specify those aspects of ageing that have a negative impact including t morbidity and potential reduction of quality of life. This would contextualize better the need to contrast those negative aspects of ageing by increasing care, in order to benefit more of the listed benefits of longer life expectancies. Could this do? | Thank you for your valuable feedback. Yes, here's a revised version:  "Longer life expectancies yield societal benefits such as enhanced workforce wisdom, intergenerational knowledge exchange, ongoing personal development, diverse innovation, and strengthened family ties. However, the challenges of an ageing population, including increased healthcare burden, declining productivity, strained social security systems, potential resource inequities, and higher demand for long-term care, threaten productivity, economic growth, and social security sustainability." | Page 2 |
| 2. | Page 3 study design: “An advantage of a cross-sectional study design was its convenience in terms of cost and time in data collection, as well as allowing the researcher to compare multiple variables simultaneously.” This paragraph could be moved as a “strength of the study” in the limits section. | Thank you for your important input. It was attended as advised and shifted from page 3 to page 15 | Page 15 |
| 3. | Page 3: study setting: could we specify better the setting? For example, is it a rural or urbanised setting? What is the main activity of the population in general, etc? This would make us contextualise better the results…. | Thank you for your valuable feedback. Here is the response;  The study took place in the Butiama and Musoma districts within Tanzania's Mara Region, predominantly rural areas known for activities such as agriculture, livestock keeping, fishing, and mining. | Page 3 |
| 4. | Page 4: the districts were selected by convenience: could you briefly specify what made their selection convenient? | Thank you for your valuable comment.  Butiama and Musoma districts were chosen conveniently for assessing OPD healthcare service utilization among older individuals due to their accessibility by considering financial constraints, transportation convenience, cooperation from local authorities, and representative status within the rural Mara region. The researcher's familiarity and existing connections further facilitated data collection and logistics. |  |
| 5. | Page 5: Independent variables: I would move the following paragraph to the statistics analysis section because it specifies how the collected variables are analysed: “To calculate the weighted household wealth index, households were scored based on their possession of consumer goods or durable assets. These scores were determined through principal component analysis (PCA) in SPSS, which reduced the variables into components using loading factors as weights, ultimately yielding the weighted wealth index. Following this, households were categorized into four equal wealth quartiles, and the scores were presented as lowest, Second, Middle, and Highest wealth index”. | Thank you for your valuable input. The issue has been addressed accordingly and moved to the analysis section of page number 6. | From pages 5 to 6. |
| 6. | Page 6: in addition to the percentage, specify the ratio: for example x widows/ total participants | Thank you for your valuable input. This matter has been addressed as follows:  Table 1 summarizes the characteristics of 415 elderly participants in the study. Seven elders were unable to participate: five rejected consent and two were mentally unfit. Data were obtained from 415 instead of 422, resulting in a response rate of 98.3%. Of the 415 participants, 171 (41.2%) were male and 244 (58.8%) were female. The mean age was 69.06 ± 6.995 years, and 271(65.3%) of the sample were aged ≤70 years. Most elders were widowed 145 (34.9%) and completed primary school education 283(68.2%). Most households 282(68.0%) had more than three family members. Most of the study participants, 269(64.8%), were engaged in unskilled labour. Most of the study participants, 254(61.2%) had monthly income of less than 100,000 Tzs (40USD). About three-quarters of respondents, 312(75.2%) fell within the middle range of the wealth index. In terms of lifestyle choices, 107(25.8%) of the elders reported consuming alcohol, and 44(10.6%) reported smoking cigarettes or using other tobacco products. Additionally, only about one-third 134(32.3%) of the respondents reported engaging in physical activities at the time of the study. | Page 6 |
| 7. | Table 2: could we specify in the Table the p-value set for significant results? Could we also add a star on those values in a table that meets significance, on the p value column? | Thank you for your important input. The comment is constructive. The issue has been resolved as advised. For bivariate analysis, the P<0.25 was considered significant | Page 9 |
| 8. | Table 3: The title doesn’t match the content: it contains not only socio-economic factors, but also anagraphic factors, and risk factors like smoking and alcohol intake. Put a star on significant values. | Thank you for your valuable input: the title has been revised to “Table 3: Multivariate analysis of factors influencing OPD visits among the elderly in Butiama and Musoma districts.” And the star added to the significant values. | Page 11 |
| 9. | Table 4: could data be presented as a graph? It could provide an easier way of perceiving the frequencies of answers in a rapid visual assessment of data  Discussion: I would move the following sentence to the last part of the discussion/ conclusions “This underutilization highlights the imperative for targeted interventions and strategies aimed at improving access and utilization, thereby enhancing healthcare outcomes for this demographic.” | Thank you for your valuable input: The data with significance value has been represented in a graph as suggested. (Please refer to fig. 2)  Additionally, the suggestion to relocate the phrase "This underutilization highlights the imperative for targeted interventions and strategies aimed at improving access and utilization, thereby enhancing healthcare outcomes for this demographic" to the conclusion's final section is acknowledged and implemented accordingly. | Page 16 |
| 10. | Page 14: check the direction of the association for the variables in this sentence: “In this study, being older than 80 years was associated with declining utilization of OPD’s healthcare services among elders “ ….it should be contrary, over eighty they seem to use more…..it is the 70-80 which underutilises it. | Thank you for your feedback and concern.  During the initial bivariate analysis, it was observed that elders aged over 80 were utilizing services more frequently, whereas individuals aged 71-80 displayed a decline. However, after adjusting for variables in the multivariate analysis, it became evident that individuals over 80 were significantly 62% less likely to visit the outpatient department compared to those under 70 years old. This finding was statistically significant, with a p-value of 0.011. | Page 14 |
| 11. | The discussion is too short, and it is too narrow when it comes to the discussion of table number 4, which gives some inputs for expansion…. | Thank you for your input, which helped strengthen the discussion. The matter has been addressed, and the discussion part has been enriched and strengthened. | Page 15 |
